# Comparative analysis of dietary patterns and depression risk: significant inverse association with HEI-2015 and mediating role of BMI

**DOI:** 10.3389/fnut.2025.1680741

**Published:** 2025-11-12

**Authors:** Zicheng Wang, Lei Fang, Fachao Shi, Qin Cui, Xiaomei Zhou

**Affiliations:** 1 Department of Hyperbaric Oxygen, The Second People's Hospital of Hefei, Hefei Hospital Affiliated to Anhui Medical University, Hefei, Anhui, China; 2 Department of Geriatrics Center, Tongling People's Hospital, Tongling, Anhui, China; 3 Department of Cardiology, Maanshan People's Hospital, Maanshan Hospital Affiliated to Wannan Medical College, Maanshan, Anhui, China

**Keywords:** depression, dietary patterns, HEI-2015, body mass index, mediation analysis, SHAP analysis

## Abstract

**Objective:**

Depression is a severe global mental disorder closely associated with dietary habits. This study aimed to evaluate associations between four dietary patterns [assessed by Dietary Inflammatory Index (DII), Healthy Eating Index-2015 (HEI-2015), Dietary Index for Gut Microbiota (DI-GM), and Composite Dietary Antioxidant Index (CDAI)] and depression risk. For any dietary pattern showing significant association, we further examined whether BMI mediated this relationship.

**Methods:**

Data from the National Health and Nutrition Examination Survey (NHANES, 2007–2018) were analyzed. Four dietary indices were calculated using two 24-h dietary recalls: DII, HEI-2015, DI-GM, and CDAI. Depression severity was assessed via the Patient Health Questionnaire-9 (PHQ-9). Logistic regression and mediation analysis were employed to examine diet-depression associations and BMI’s mediating effect. For any dietary pattern showing significant association with depression, employ SHapley Additive exPlanations (SHAP) analysis to identify which specific dietary components contribute most to this association.

**Results:**

HEI-2015 showed a significant negative correlation with depression (OR = 0.99, 95% CI: 0.98–1.00, *p* = 0.002). Compared to the lowest HEI-2015 quartile (Q1), the highest quartile (Q4) had significantly reduced depression risk (OR = 0.66, 95% CI: 0.50–0.87, *p* = 0.003). No significant associations were observed for DII, DI-GM, or CDAI. Mediation analysis revealed BMI partially mediated the HEI-2015–depression relationship (mediation proportion = 6.39%, *p* < 0.0001). SHAP analysis identified added sugars, whole fruits, and saturated fats as key HEI-2015 components: added sugars and whole fruits reduced depression risk, while saturated fats increased it.

**Conclusion:**

This study confirms a significant inverse association between HEI-2015 and depression risk, with BMI acting as a partial mediator. Reducing intake of added sugars and saturated fats while increasing whole fruits consumption may mitigate depression risk.

## 1 Introduction

Depression stands as one of the most challenging mental health issues of the 21st century, affecting global populations at an alarming rate. According to the latest WHO data, over 350 million people worldwide suffer from depression, representing a nearly 20% increase over the past decade (1). Depression is not only a leading cause of global disability but also coexists with various chronic diseases, significantly reducing patients’ quality of life, increasing suicide risk, and creating substantial socioeconomic burdens estimated at over $1 trillion annually in economic losses. Facing this growing public health challenge, identifying feasible, economical, and easily implementable prevention strategies has become particularly urgent (2). Research suggests that dietary habits affect mental health through several biological pathways, such as neurotransmitter regulation, gut microbiota balance, and reduced systemic inflammation, all of which play important roles in the development of depression (3, 4).

Different dietary patterns impact depression through a variety of mechanisms. The DII, developed to measure the inflammatory potential of diets, is widely used in research exploring links between diet-induced inflammation and mental health (5). Chronic inflammation is recognized as a key contributor to depression; higher DII scores are associated with elevated inflammatory markers, which may promote depressive symptoms by increasing neuroinflammation or activating central immune pathways (6). On the other hand, the HEI-2015 evaluates overall diet quality, with higher scores reflecting greater adherence to nutritional guidelines (7). Diets with higher HEI-2015 scores rich in fruits, vegetables, and whole grains, and lower in added sugars and sodium have been shown to lower depression risk by reducing systemic inflammation, improving metabolic health, and supporting neural function (8, 9). Recent studies also emphasize the influence of gut microbiota on depression, mediated by dietary habits. The DI-GM assesses intake of prebiotic and probiotic foods, as well as components like fiber and polyphenols that modulate gut bacteria and alleviate depressive symptoms through the gut-brain axis (10, 11). Additionally, the CDAI, which estimates overall dietary antioxidant intake, may protect neural health by reducing oxidative stress—a known factor in the development of depression (12).

Within this nutritional framework, weight status emerges as a critical factor connecting dietary patterns to depression risk. The bidirectional relationship between obesity and depression has attracted increasing research attention as evidence accumulates that these conditions share underlying biological mechanisms and mutually reinforce each other. Research indicates that excess adiposity may increase depression vulnerability through pathways involving chronic low-grade inflammation and metabolic dysregulation (13, 14) processes that notably overlap with the inflammatory and metabolic effects of poor dietary patterns. Simultaneously, depressive symptoms such as diminished motivation, disrupted sleep, and emotional eating can promote unhealthy dietary behaviors and subsequent weight gain, potentially creating a self-perpetuating cycle (15). In this complex interplay, body mass index (BMI) appears to function as a significant mediator in the diet-depression relationship, helping to elucidate mechanistic pathways. For example, dietary patterns scoring high on HEI-2015 may ameliorate depressive symptoms partially by promoting healthy weight maintenance and metabolic homeostasis (8), whereas pro-inflammatory diets with elevated DII scores may exacerbate both systemic inflammation and psychological distress partly through their association with increased BMI (16).

Exploring the effects of various dietary patterns on depression risk has important clinical relevance. Early identification of individuals with poor dietary habits, alongside interventions to improve dietary quality (such as boosting HEI-2015 scores), could serve as an effective strategy for depression prevention (17). The mediation analysis in this study is based on multiple theoretical pathways connecting dietary patterns, BMI, and depression. Dietary patterns may influence BMI through energy balance regulation, metabolic programming, gut-brain axis modulation, and inflammatory pathways (18–20). Simultaneously, BMI may affect depression risk through biological mechanisms (chronic inflammation, neuroendocrine alterations) and psychosocial factors (weight stigma, body dissatisfaction) (21–23). Therefore, BMI may serve as a mediator in the diet-depression relationship, suggesting that diet quality might partially influence depression risk through its effects on weight status, while we also acknowledge that diet may impact depression through other direct pathways (such as nutrient-specific neural mechanisms) (24, 25).

This study analyzes the associations among dietary patterns, BMI, and depression using the NHANES database. Utilizing machine learning methods such as SHAP analysis, we systematically assessed the influence of specific dietary components on depression outcomes. However, few studies have simultaneously examined multiple dietary patterns in relation to depression, and even fewer have investigated potential mediating mechanisms. Furthermore, the comparative utility of different dietary indices for predicting depression risk remains unclear. This study aims to address these gaps by examining four established dietary patterns and exploring potential mediating pathways.

## 2 Methods

### 2.1 Study population

NHANES is a nationwide cross-sectional survey conducted by the Centers for Disease Control and Prevention, systematically collecting health information from U.S. residents using a multi-stage stratified sampling approach. This ongoing project has received approval from the National Center for Health Statistics Ethics Review Board, and all participants provided written informed consent before enrollment. Since this study utilizes de-identified, publicly available data and involves no new interventions, it was exempted from additional ethical review by the Ethics Committee of Anhui Provincial Hospital.

**Figure 1:**
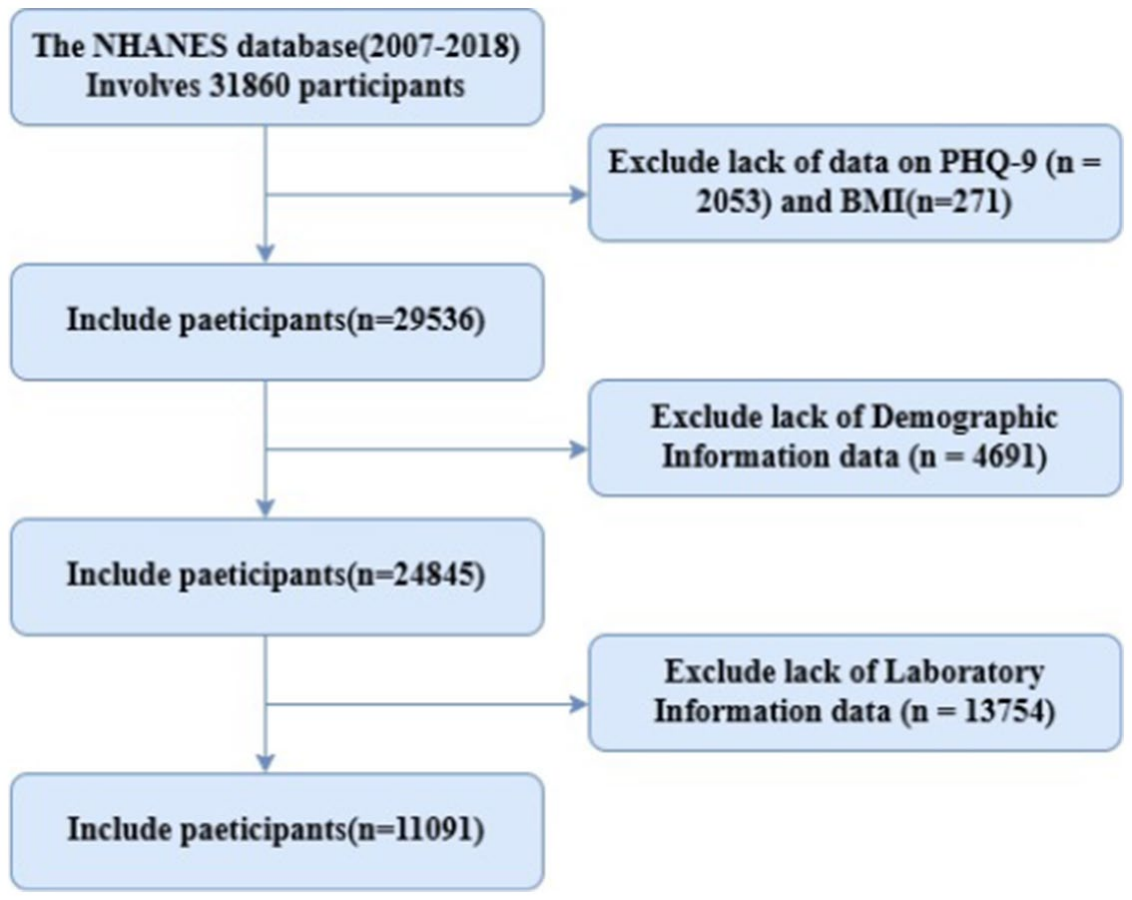
Study flow chart.

This research draws on data from six NHANES cycles (2007–2018), initially including 31,860 participants aged 18 years or older. Depression was assessed using the Patient Health Questionnaire-9 (PHQ-9), a well-validated self-administered screening tool widely used in both clinical and research settings to measure depression severity. The PHQ-9 consists of nine items corresponding to the nine DSM-IV diagnostic criteria for major depressive disorder. Each item asks respondents to rate the frequency of specific depressive symptoms over the past 2 weeks on a four-point Likert scale: 0 (not at all), 1 (several days), 2 (more than half the days), and 3 (nearly every day). Total scores range from 0 to 27, with higher scores indicating greater depression severity (26–29). In accordance with established clinical guidelines and previous NHANES-based studies, we defined depression using the following classification: a PHQ-9 total score ≥10 was classified as clinically significant depression (moderate to severe), while scores <10 were classified as minimal to mild depressive symptoms. This cutoff of ≥10 has been extensively validated, demonstrating a sensitivity of 88% and specificity of 88% for major depressive disorder when compared to structured clinical interviews. Additionally, we conducted sensitivity analyses using alternative PHQ-9 cutoff points (≥5 for mild depression, ≥15 for moderately severe depression) to ensure the robustness of our findings. For the machine learning analysis, we retained the continuous PHQ-9 score to preserve the full spectrum of depressive symptomatology and maximize statistical power. The internal consistency reliability of the PHQ-9 in our study population was excellent (Cronbach’s *α* = 0.84), confirming the psychometric robustness of this measure in our sample. Rigorous data cleaning was performed, excluding 2,053 participants missing PHQ-9 data, 271 missing BMI, and those lacking complete information on marital status (*n* = 27), education (*n* = 13), family income-to-poverty ratio (PIR, *n* = 2,484), smoking (*n* = 9), alcohol use (*n* = 876), COPD (*n* = 1,006), hypertension (*n* = 1), diabetes (*n* = 273), and cardiovascular disease (*n* = 2). Participants with missing laboratory results were also excluded, encompassing renal function (eGFR, *n* = 1,144), blood glucose (*n* = 12,269), HbA1c (*n* = 23), liver enzymes (ALT/AST, *n* = 12), and metabolic indicators (uric acid/LDL, *n* = 306). Ultimately, 11,091 eligible participants were included, as illustrated in the flowchart (Figure 1).

### 2.2 Dietary indices

This study collected dietary data using NHANES’s standardized 24-h dietary recall method. The first recall interview was conducted in a Mobile Examination Center, followed by a second assessment by telephone 3 to 10 days later. Dietary intake was calculated as the average of both recalls; if only one was available, the first recall data was used. Nutritional composition for all foods and beverages was determined using USDA Food Patterns Equivalent Database categories, supplemented with the Food and Nutrition Database for Dietary Studies for energy and nutrient estimations (30, 31).

The 24-h dietary recall data were used to calculate four distinct dietary indices, each with unique conceptual frameworks, components, and scoring methodologies: dietary Inflammatory Index (DII) evaluates the inflammatory potential of diets based on 26 nutrients, with positive scores indicating pro-inflammatory effects and negative scores indicating anti-inflammatory effects (32, 33). The DII specifically targets the inflammatory pathway, which is one potential mechanism linking diet and depression. Healthy Eating Index-2015 (HEI-2015) assesses overall diet quality based on adherence to the 2015–2020 Dietary Guidelines for Americans. It consists of 13 components (9 adequacy components: Total Fruits, Whole Fruits, Total Vegetables, Greens and Beans, Whole Grains, Dairy, Total Protein Foods, Seafood and Plant Proteins, and Fatty Acids; and 4 moderation components: Refined Grains, Sodium, Added Sugars, and Saturated Fats). Each component is scored per 1,000 calories, with total scores ranging from 0 to 100. Higher scores indicate greater adherence to dietary guidelines (34, 35). Unlike other indices, HEI-2015 provides a comprehensive evaluation of diet quality that balances both nutrient adequacy and moderation components. Dietary Index for Gut Microbiota (DI-GM) was calculated based on the Kase standard, covering nine beneficial components (including avocados, whole grains, fruits, vegetables, nuts, beans, fish, yogurt, and coffee) and four detrimental components (red meat, processed meat, alcohol, and refined grains) that specifically influence gut microbiota composition. Green tea was excluded due to lack of data (36). This index focuses exclusively on the gut microbiota pathway between diet and health outcomes. Composite Dietary Antioxidant Index (CDAI) measures dietary antioxidant capacity by incorporating six micronutrients with known antioxidant properties: zinc, selenium, carotenoids, and vitamins A, C, and E, following Wright’s methodology (37). The CDAI specifically targets the oxidative stress pathway as a potential mechanism in depression pathophysiology.

### 2.3 Covariates

Covariates in this study included demographic factors (age, sex, race, education, marital status), medical history (hypertension, diabetes, cardiovascular disease, chronic obstructive pulmonary disease), lifestyle factors (smoking, alcohol use, leisure physical activity), and socioeconomic status (poverty-income ratio, PIR). Variable classifications were as follows: race (Non-Hispanic White, Non-Hispanic Black, Mexican American, Other); PIR (<1.3, 1.3–3.5, >3.5); marital status (Married, Divorced, Unmarried, Other); smoking (Never: <100 cigarettes in a lifetime; Former: ≥100 cigarettes, quit; Current: ≥100 cigarettes, still smoking); alcohol consumption (Never: <12 times ever; Former: ≥12 times/year, quit; Light: less than moderate/heavy; Moderate: women 2 drinks/day or binge drinking on ≥2 days/month, men—3 drinks/day; Heavy: women ≥3 drinks/day or binge ≥5 days/month, men ≥4 drinks/day); BMI (Normal: <25 kg/m^2^, Overweight: 25–30, Obese: ≥30). Laboratory measures included metabolic indicators (blood glucose, HbA1c, energy intake), kidney function (eGFR, creatinine, uric acid, BUN), liver function (ALT, AST, albumin), and lipid profile (TC, TG, HDL, LDL).

### 2.4 Statistical analysis

All statistical analyses were conducted using R version 4.3.2, following NHANES analytical guidelines (31) to account for the complex sampling design and appropriate weights (WTMEC2YR/6). Continuous variables were expressed as means (standard error), while categorical variables were reported as proportions (standard error). Group differences were evaluated using analysis of variance or chi-square tests, as appropriate.

Our analytical approach consisted of three sequential steps: First, we evaluated associations between each dietary pattern and depression using multivariable logistic regression. Second, for dietary patterns showing significant associations, we conducted mediation analysis to examine BMI’s potential mediating role. Third, for the most strongly associated dietary pattern, we performed additional component analysis using machine learning approaches. It is worth noting that our mediation analysis of BMI was conducted only after establishing a significant association between HEI-2015 and depression, following appropriate statistical practice for mediation analysis where the independent variable must first show association with the dependent variable.

Dietary patterns were divided into quartiles (Q1–Q4) and their association with depression risk was evaluated using weighted multivariable logistic regression models. Potential confounders were selected based on two complementary approaches: (1) established risk factors for depression identified in prior literature; (2) variables demonstrating associations with both dietary patterns and depression in our preliminary analyses. Based on these methods, we identify three statistical models. Model 1 was unadjusted; Model 2 adjusted for age, race, and gender; and Model 3 further adjusted for metabolic indicators (blood glucose, triglycerides, LDL), comorbidities (hypertension, diabetes, COPD, cardiovascular disease), and PIR. These confounders were consistently applied across all analytical models to ensure comparability of results. A restricted cubic spline (RCS) analysis was performed to assess dose–response relationships, and subgroup analyses were conducted by age (<60/≥60 years), gender, race (Mexican American, non-Hispanic White, non-Hispanic Black, Other), PIR (<1.3, 1.3–3.5, >3.5), and presence of hypertension, diabetes, COPD, or CVD.

To evaluate whether BMI mediates the relationship between dietary patterns and depression, we conducted formal mediation analysis using the counterfactual framework approach. For this analysis, HEI-2015 was treated as continuous exposure variables, BMI as a continuous mediator, and depression as a binary outcome (PHQ-9 ≥ 10). This approach was selected to preserve maximum statistical power while maintaining clinical interpretability of the depression outcome.

We implemented the mediation analysis using the ‘mediation’ package in R, which employs the following sequential models: Mediator model: BMI (continuous) ~ Dietary index (continuous) + Confounders; Outcome model: Depression (binary) ~ Dietary index (continuous) + BMI (continuous) + Confounders. Both models were adjusted for the same set of confounders used in our main analyses: age, race, gender, blood glucose, triglycerides, LDL, hypertension, diabetes, COPD, cardiovascular disease and PIR.

We calculated the following mediation parameters: Natural direct effect: Effect of dietary patterns on depression not mediated through BMI; Natural indirect effect: Effect of dietary patterns on depression mediated through BMI; Total effect: Sum of direct and indirect effects; Proportion mediated: Percentage of the total effect mediated through BMI. Statistical inference was based on 5,000 bootstrap resamples to derive 95% confidence intervals for all mediation parameters. Sensitivity analyses were performed to assess the robustness of our findings to potential unmeasured confounding using the E-value approach.

Five machine learning models were employed to assess the impact of dietary patterns on depression risk: MLP, DT, XGBoost, LR, and RF. For dietary patterns demonstrating significant associations with depression in our primary analysis, we conducted SHAP (SHapley Additive exPlanations) analysis to identify which specific dietary components contributed most substantially to the observed association. This machine learning approach was applied only to significantly associated dietary patterns for two reasons: First, SHAP analysis is most meaningfully applied when a meaningful association exists between the overall index and the outcome. Second, applying complex machine learning methods to non-significant associations would constitute unnecessary data mining without clear theoretical justification and would increase the risk of false positive findings due to multiple testing. The weighted average of all SHAP values reflects the overall importance of features, visualized using global feature importance and bee swarm plots.

## 3 Results

### 3.1 Characteristics

A total of 11,091 participants were included in this study (49.34% male), with a mean age of 47.74 ± 0.29 years. Among them, 957 individuals (8.63%) were identified with depression. As summarized in Table 1, compared to the non-depressed group, those with depression were more likely to be unmarried, divorced, or cohabiting (*p* < 0.05), and had a higher proportion of females. Depressed participants also had lower education levels, lower PIR, and less frequent engagement in recreational activities, while rates of current smoking and moderate to heavy drinking were higher (*p* < 0.01). Additionally, they exhibited significantly higher rates of comorbidities including hypertension, diabetes, COPD, and cardiovascular disease (*p* < 0.001).

**Table 1:** Comparison of general data between depressed and non-depressed patients.

Variables	Total	Non-Depression	Depression	*p*-value
Age, mean (SE)	47.74 (0.29)	47.74 (0.31)	47.76 (0.63)	0.98
Creatinine, mean (SE)	77.80 (0.36)	77.82 (0.35)	77.54 (2.40)	0.91
UA, mean (SE)	326.67 (1.24)	327.21 (1.27)	319.96 (3.41)	0.04
BUN, mean (SE)	4.87 (0.03)	4.89 (0.03)	4.62 (0.08)	0.001
Glucose, mean (SE)	5.91 (0.02)	5.89 (0.03)	6.17 (0.08)	0.002
eGFR, mean (SE)	94.73 (0.37)	94.71 (0.39)	95.05 (0.89)	0.72
BMI, mean (SE)	29.07 (0.11)	28.94 (0.11)	30.63 (0.28)	< 0.0001
HbA1c, mean (SE)	5.62 (0.01)	5.61 (0.01)	5.79 (0.04)	< 0.001
ALT, mean (SE)	25.14 (0.21)	25.08 (0.22)	25.92 (0.78)	0.32
AST, mean (SE)	25.14 (0.19)	25.03 (0.19)	26.53 (1.11)	0.19
TG, mean (SE)	1.31 (0.01)	1.30 (0.01)	1.49 (0.04)	< 0.0001
TC, mean (SE)	4.96 (0.02)	4.96 (0.02)	5.03 (0.05)	0.15
LDL, mean (SE)	2.95 (0.01)	2.95 (0.01)	2.98 (0.04)	0.48
HDL, mean (SE)	1.41 (0.01)	1.41 (0.01)	1.37 (0.02)	0.02
Albumin, mean (SE)	4.25 (0.01)	4.25 (0.01)	4.15 (0.01)	< 0.0001
Energy, mean (SE)	2180.29 (11.73)	2188.67 (12.30)	2077.66 (50.56)	0.04
DII, mean (SE)	1.41 (0.04)	1.37 (0.04)	1.93 (0.09)	< 0.0001
HEI-2015, mean (SE)	50.65 (0.27)	50.92 (0.29)	47.31 (0.50)	< 0.0001
CADI, mean (SE)	0.79 (0.07)	0.85 (0.07)	0.14 (0.19)	< 0.001
DI-GM, mean (SE)	4.71 (0.03)	4.72 (0.03)	4.57 (0.06)	0.05
Sex,% (SE)				<0.0001
Male	49.34 (0.02)	50.36 (0.55)	36.89 (2.16)	
Female	50.66 (0.02)	49.64 (0.55)	63.11 (2.16)	
Race, % (SE)				0.04
Mexican American	7.97 (0.01)	8.03 (0.71)	7.26 (1.13)	
Non-Hispanic Black	9.73 (0.01)	9.52 (0.70)	12.33 (1.18)	
Non-Hispanic White	70.17 (0.03)	70.44 (1.40)	66.81 (2.23)	
Other	12.12 (0.01)	12.00 (0.70)	13.60 (1.51)	
Marital, % (SE)				<0.0001
Married	56.03 (0.02)	57.49 (0.99)	38.14 (2.12)	
Never Married	17.71 (0.01)	17.52 (0.74)	20.02 (1.85)	
Divorced	10.61 (0.01)	9.93 (0.45)	18.91 (1.63)	
Unmarried but have/had partner	15.66 (0.01)	15.07 (0.55)	22.92 (1.52)	
Education, % (SE)				<0.0001
Less than high School	14.59 (0.01)	13.74 (0.69)	25.03 (1.72)	
High school or equivalent	22.75 (0.01)	22.43 (0.80)	26.76 (1.76)	
College or above	62.65 (0.02)	63.83 (1.16)	48.21 (2.30)	
Smoke, % (SE)				<0.0001
Never	55.30 (0.02)	56.73 (0.86)	37.89 (1.95)	
Former	25.80 (0.01)	26.05 (0.78)	22.72 (2.00)	
Now	18.89 (0.01)	17.22 (0.64)	39.39 (2.11)	
Alcohol, % (SE)				<0.0001
Never	10.08 (0.01)	10.13 (0.59)	9.49 (0.79)	
Former	12.69 (0.01)	12.23 (0.54)	18.30 (1.82)	
Mild	38.49 (0.01)	39.40 (0.89)	27.35 (2.24)	
Moderate	17.84 (0.01)	17.93 (0.53)	16.75 (1.71)	
Heavy	20.90 (0.01)	20.31 (0.62)	28.10 (1.77)	
Diabetes, % (SE)				<0.001
Yes	16.00 (0.01)	15.49 (0.61)	22.31 (1.53)	
No	66.50 (0.02)	67.05 (0.83)	59.79 (1.89)	
Borderline	17.49 (0.01)	17.46 (0.56)	17.90 (1.53)	
Hypertension, % (SE)				<0.001
Yes	38.16 (0.01)	37.38 (0.82)	47.66 (2.52)	
No	61.84 (0.02)	62.62 (0.82)	52.34 (2.52)	
PIR, % (SE)				<0.0001
<1.3	20.74 (0.01)	19.05 (0.79)	41.50 (2.28)	
1.3–3.5	35.67 (0.01)	35.74 (0.85)	34.84 (2.16)	
>3.5	43.59 (0.02)	45.22 (1.19)	23.66 (2.17)	
Recreational Activity, % (SE)				<0.0001
Yes	54.62 (0.02)	56.28 (1.02)	34.38 (2.35)	
No	45.38 (0.02)	43.72 (1.02)	65.62 (2.35)	
COPD, % (SE)				<0.001
Yes	5.02 (0.00)	4.77 (0.33)	8.07 (1.04)	
No	94.98 (0.03)	95.23 (0.33)	91.93 (1.04)	
CVD, % (SE)				<0.0001
Yes	8.97 (0.00)	8.36 (0.39)	16.49 (1.46)	
No	91.03 (0.03)	91.64 (0.39)	83.51 (1.46)	
DIIQ, % (SE)				<0.0001
Q1	27.46 (0.01)	28.05 (0.79)	20.19 (1.73)	
Q2	25.58 (0.01)	25.83 (0.62)	22.46 (1.66)	
Q3	24.34 (0.01)	24.40 (0.63)	23.65 (1.91)	
Q4	22.62 (0.01)	21.72 (0.83)	33.70 (2.00)	
HEI-2015Q, % (SE)				<0.0001
Q1	25.18 (0.01)	24.67 (0.75)	31.52 (2.05)	
Q2	25.37 (0.01)	25.21 (0.67)	27.32 (2.34)	
Q3	24.99 (0.01)	24.89 (0.63)	26.22 (2.15)	
Q4	24.46 (0.01)	25.24 (0.82)	14.94 (1.39)	
CDAIQ, % (SE)				<0.0001
Q1	21.66 (0.01)	20.85 (0.68)	31.51 (1.80)	
Q2	24.20 (0.01)	24.21 (0.52)	24.01 (2.13)	
Q3	26.86 (0.01)	27.31 (0.65)	21.28 (1.62)	
Q4	27.29 (0.01)	27.62 (0.68)	23.20 (1.91)	
DI-GMQ, % (SE)				0.03
Q1	46.47 (0.01)	46.04 (0.84)	51.65 (2.06)	
Q2	22.97 (0.01)	22.97 (0.66)	23.00 (1.57)	
Q3	16.52 (0.01)	16.62 (0.60)	15.27 (1.67)	
Q4	14.04 (0.01)	14.36 (0.61)	10.07 (1.43)	

Date are presented as mean (SE) or %(SE); ALT, Alanine aminotransferase; AST, Aspartate aminotransferase; BMI, Body mass index; BUN, Blood urea nitrogen; CVD, Cardiovascular disorders; COPD, Chronic obstructive pulmonary disease; DII, Dietary Inflammatory Index; CDAI, Composite Dietary Antioxidant Index; DIGM, Dietary Index for Gut Microbiota; eGFR, Estimated glomerular filtration rate; HbA1c, Glycosylated hemoglobin; HEI-2015, Healthy Eating Index 2015; HDL, High density lipoprotein; LDL, Low density lipoprotein; PIR, Poverty income ratio; TG, Triglyceride; TC, Total cholesterol; UA, Uric acid.

### 3.2 Association of different dietary patterns with risk of depression

**Figure 2:**
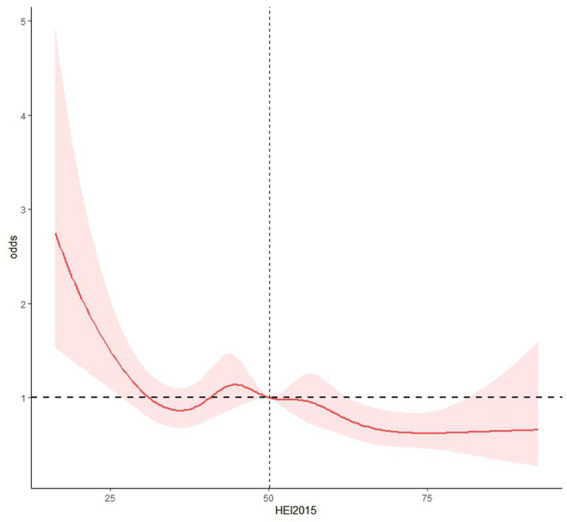
Restricted cubic spline analysis showed a non-linear dose–response relationship between HEI-2015 and risk of depression.

As shown in Table 2. Among the four dietary indices evaluated, after adjusting for confounders, each 1-point increase in HEI-2015 was associated with a 1% reduction in depression risk (OR = 0.99, 95% CI: 0.98–1.00, *p* = 0.002). Participants in the highest HEI-2015 quartile (Q4) had a 34% lower risk of depression compared to those in the lowest quartile (Q1) (OR = 0.66, 95% CI: 0.50–0.87, *p* = 0.003). By contrast, no statistically significant associations were observed for DII, DI-GM, or CDAI after full adjustment for covariates (*p* > 0.05). Consequently, we focused subsequent mediation and component analyses on HEI-2015. Restricted cubic spline analysis with four knots placed at the 5th, 35th, 65th, and 95th percentiles revealed a nonlinear dose–response relationship between HEI-2015 scores and depression risk (P for non-linearity = 0.016) (Figure 2). The association was characterized by a steeper reduction in depression risk at lower HEI-2015 scores, with a plateau effect observed at higher scores, suggesting diminishing marginal benefits of further diet quality improvements beyond this threshold.

**Table 2:** The analysis of the correlation between different dietary patterns and depression.

Variables	Model 1		Model 2		Model 3	
	OR (95%CI)	*p*	OR (95%CI)	*p*	OR (95%CI)	*p*
DII	1.18 (1.12, 1.25)	<0.0001	1.15 (1.09, 1.22)	<0.0001	1.05 (1.00, 1.11)	0.06
DIIQ						
Q1	Ref	Ref	Ref	Ref	Ref	Ref
Q2	1.21 (0.95, 1.54)	0.12	1.15 (0.90, 1.47)	0.25	1.04 (0.82, 1.33)	0.73
Q3	1.35 (1.00, 1.81)	0.05	1.23 (0.91,1.66)	0.18	0.95 (0.71, 1.27)	0.72
Q4	2.16 (1.68, 2.76)	<0.0001	1.89 (1.46, 2.43)	<0.0001	1.26 (0.99, 1.62)	0.06
P for trend	<0.0001		<0.0001		0.119	
HEI-2015	0.98 (0.97, 0.99)	<0.0001	0.98 (0.97, 0.98)	<0.0001	0.99 (0.98, 1.00)	0.002
HEI-2015Q						
Q1	Ref	Ref	Ref	Ref	Ref	Ref
Q2	0.85 (0.65, 1.11)	0.23	0.85 (0.65, 1.11)	0.22	0.98 (0.73, 1.30)	0.86
Q3	0.82 (0.63, 1.08)	0.17	0.80 (0.61, 1.04)	0.09	1.00 (0.74, 1.34)	0.99
Q4	0.46 (0.36, 0.59)	<0.0001	0.43 (0.34, 0.56)	<0.0001	0.66 (0.50, 0.87)	0.003
P for trend	<0.0001		<0.0001		0.02	
CDAI	0.95 (0.92, 0.98)		0.96 (0.93, 0.99)	0.004	0.99 (0.96, 1.01)	0.34
CDAIQ						
Q1	Ref	Ref	Ref	Ref	Ref	Ref
Q2	0.66 (0.50, 0.86)	0.002	0.68 (0.52, 0.89)	0.01	0.83 (0.63, 1.09)	0.18
Q3	0.52 (0.41, 0.64)	<0.0001	0.54 (0.43, 0.67)	<0.0001	0.73 (0.58, 0.93)	0.01
Q4	0.56 (0.44, 0.70)	<0.0001	0.59 (0.46, 0.74)	<0.0001	0.82 (0.65, 1.03)	0.08
P for trend	<0.0001		<0.0001		0.046	
DI-GM	0.95 (0.90, 1.00)	0.05	0.94 (0.89, 1.00)	0.03	1.01 (0.95, 1.07)	0.82
DI-GMQ						
Q1	Ref	Ref	Ref	Ref	Ref	Ref
Q2	0.89 (0.72, 1.11)	0.29	0.88 (0.71, 1.09)	0.25	0.95 (0.76, 1.19)	0.67
Q3	0.82 (0.61, 1.11)	0.19	0.82 (0.60, 1.12)	0.21	1.02 (0.73, 1.43)	0.91
Q4	0.62 (0.45, 0.87)	0.01	0.61 (0.44, 0.84)	0.003	0.83 (0.59, 1.17)	0.28
P for trend	0.003		0.003		0.445	

HR, hazard ratio; CI, confidence interval; Ref, reference.

Model 1: No adjustments made; Model 2: Adjusted for age, sex, Race; Model 3: Adjusted for age, sex, race, hypertension, diabetes, COPD, CVD, PIR, TG, LDL and glucose.

### 3.3 Mediation analysis and subgroup analysis

**Figure 3:**
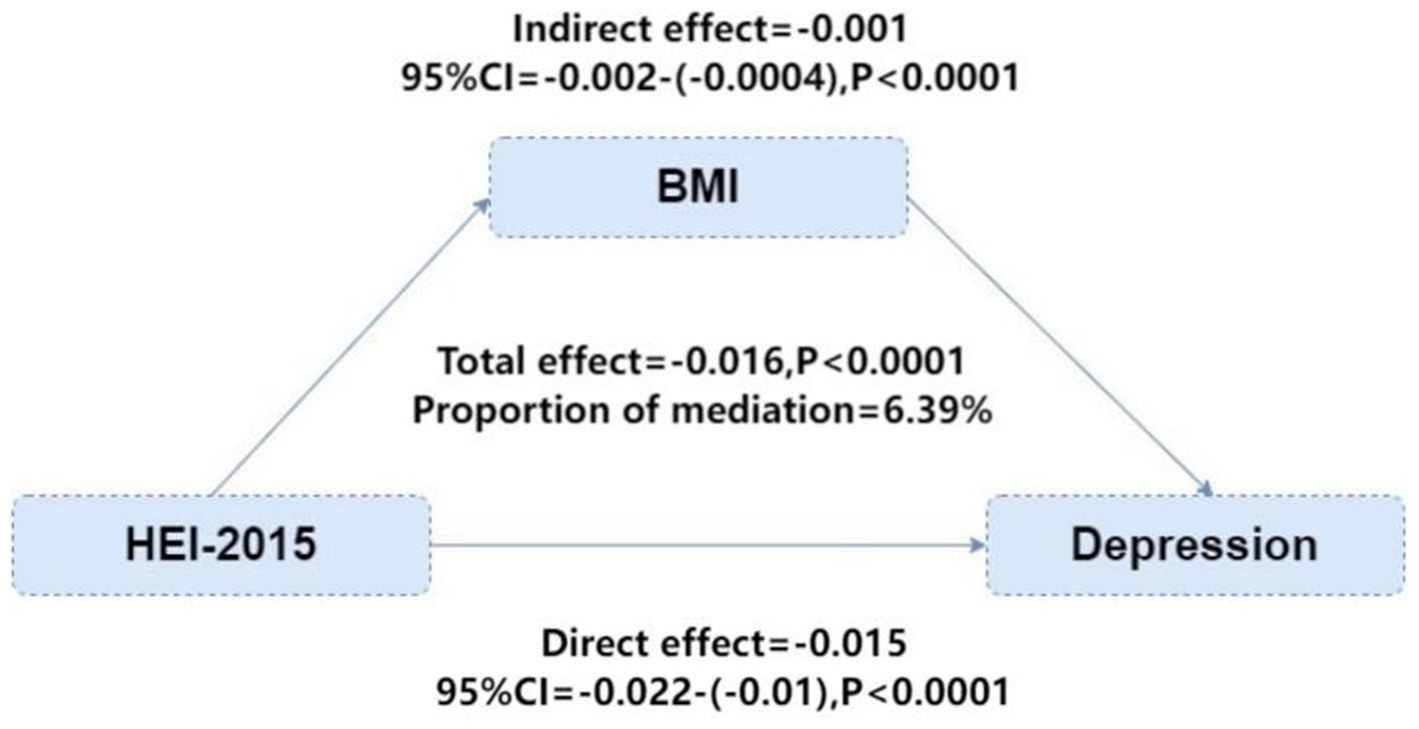
Mediation effect analysis of BMI between HEI-2015 and risk of depression.

**Figure 4:**
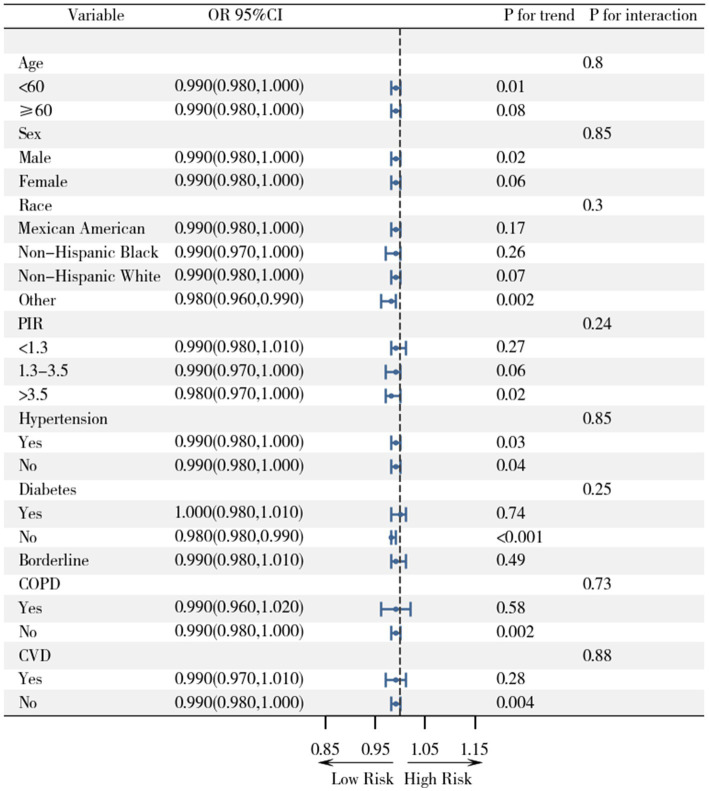
Subgroup analysis of the association between dietary patterns and depression by demographic characteristics.

In our mediation analysis examining whether BMI mediates the relationship between diet quality and depression, HEI-2015 was modeled as a continuous variable to maximize statistical power and capture the full spectrum of diet quality. The continuous HEI-2015 scores were used in both the mediator model (with BMI as outcome) and the outcome model (with depression as outcome). This approach allowed us to quantify the direct and indirect effects associated with each unit increase in HEI-2015 score. Mediation analysis indicated that BMI partially mediates the relationship between HEI-2015 and depression risk, with the indirect effect accounting for 6.39% of the total effect (*β* = −0.016, *p* < 0.0001; Figure 3). Subgroup analyses (Figure 4) showed that the protective association of HEI-2015 is consistent across all age, gender, race, PIR, and comorbidity groups, with no significant interactions observed (*p* > 0.05).

### 3.4 Machine learning model selection and SHAP analysis

**Figure 5:**
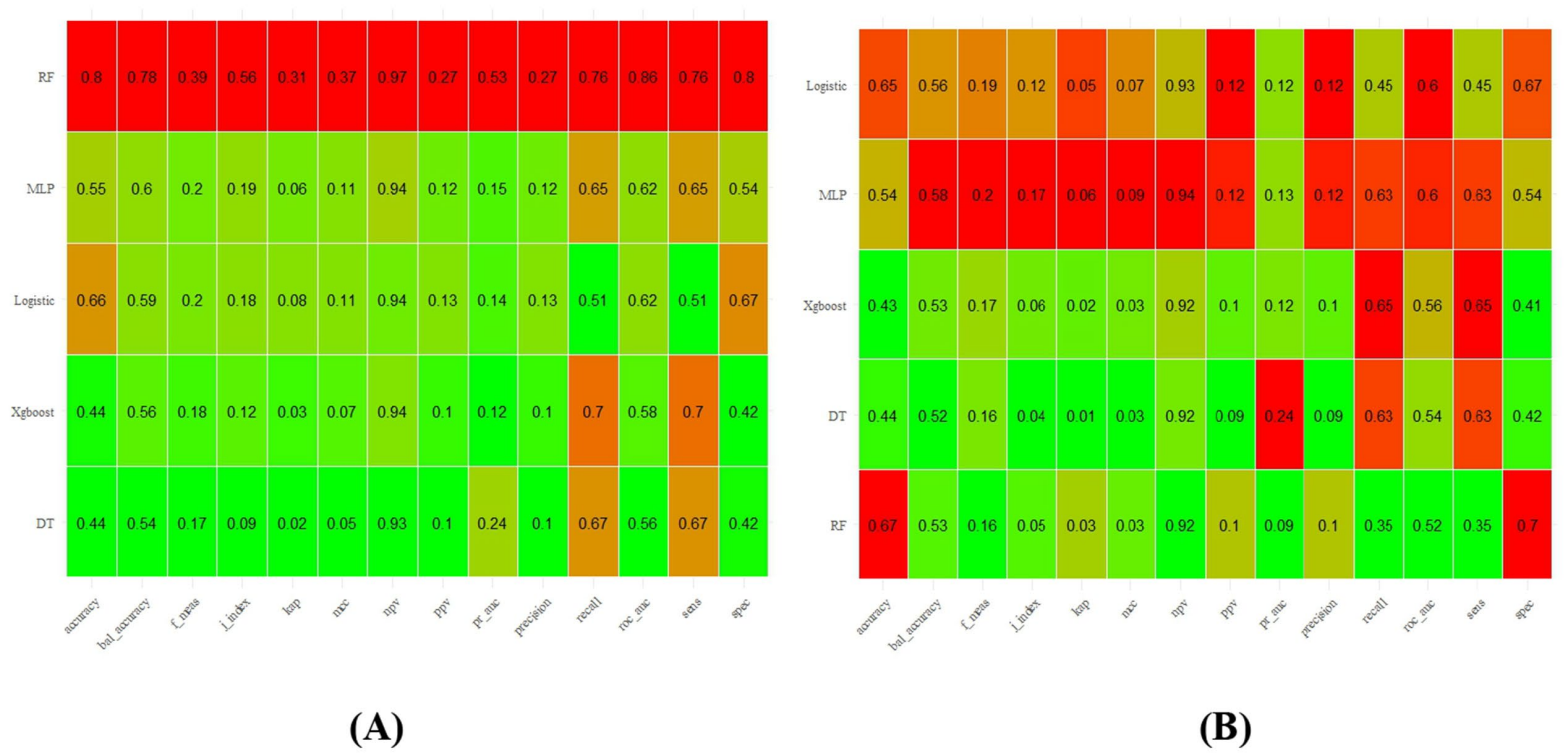
Comparison between machine learning training set and validation set; **(A)** training set, **(B)** validation set.

**Figure 6:**
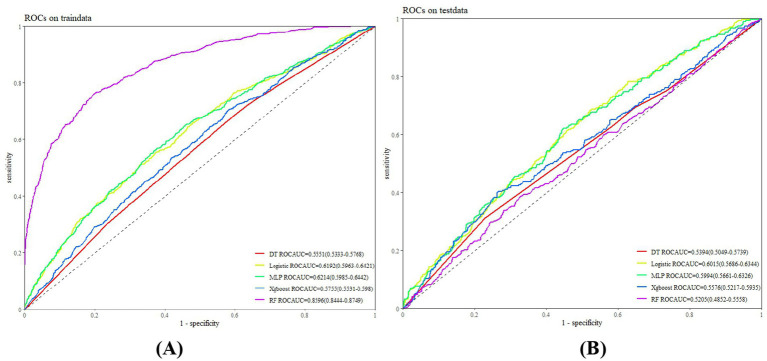
Comparison of receiver operating characteristic curves between machine learning training set and validation set;**(A)**training set,**(B)** validation set.

The HEI-2015 components dataset was randomly split into training and test sets at a 7:3 ratio, with the training set used for model development and parameter tuning, and the test set reserved for performance evaluation. As shown in Figure 5, the RF model achieved the highest performance in the training set (AUC = 0.86, accuracy = 0.80, sensitivity = 0.76, specificity = 0.80, F1 = 0.39), followed by the MLP (AUC = 0.62, accuracy = 0.55, sensitivity = 0.65, specificity = 0.54, F1 = 0.20). On the testing set, MLP’s performance remained stable (AUC = 0.60, accuracy = 0.54, sensitivity = 0.63, specificity = 0.54, F1 = 0.20), while RF showed signs of overfitting (AUC = 0.52, accuracy = 0.67, sensitivity = 0.35, specificity = 0.70, F1 = 0.16), making it unsuitable for final model selection. Overall, the MLP model demonstrated robust performance, as illustrated by the ROC curves in Figure 6.

**Figure 7:**
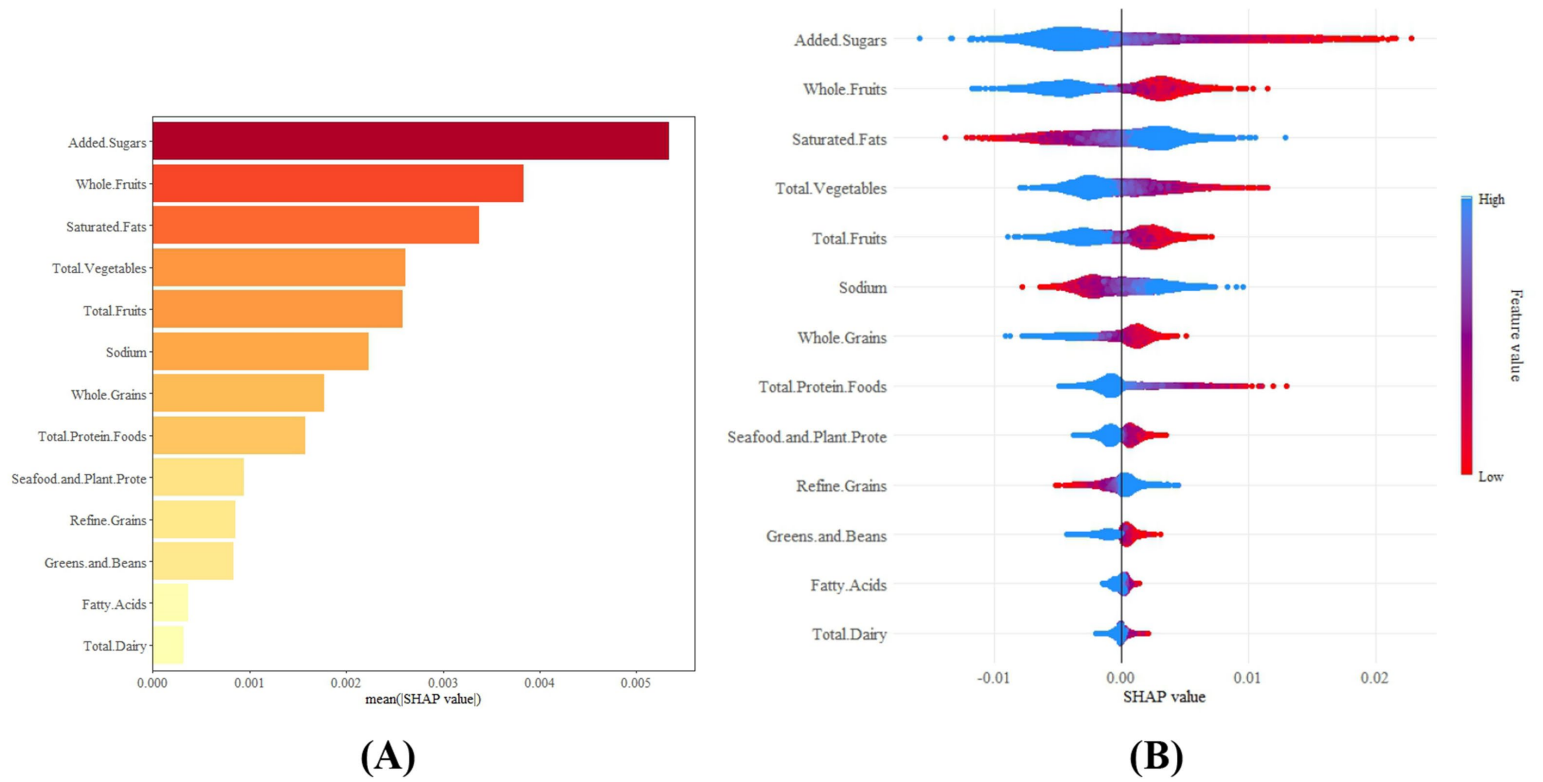
Feature-ranking plots **(A)** and summary plots **(B)** of MLP for predicting depression risk.

Given that HEI-2015 was the only dietary index showing significant association with depression after full adjustment, we focused our SHAP analysis exclusively on its components. This targeted approach allowed us to identify the most influential dietary elements within the context of a meaningful overall association, avoiding unnecessary multiple testing and data mining of non-significant relationships (Figures 7A,B). SHAP values above zero indicated an increased risk, with higher values reflecting greater impact. The analysis identified added sugars, whole fruits, and saturated fats as key factors. Specifically, higher intake of added sugars and whole fruits was linked to a reduced risk of depression, while saturated fat intake was associated with elevated risk. These findings support recommendations to reduce saturated fat and added sugar intake, while promoting consumption of whole fruits to lower depression risk.

## 4 Discussion

This study examined the relationship between dietary patterns and depression, with particular attention to the mediating role of BMI in the association between HEI-2015 and depression. Four dietary indices were evaluated: DII, HEI-2015, DI-GM, and CDAI. Higher HEI-2015 scores were significantly linked to a lower risk of depression (OR = 0.66, 95% CI: 0.50–0.87), with BMI mediating 6.39% of this effect, suggesting that healthier diets may reduce depression risk by improving obesity-related inflammation and metabolic dysfunction (38, 39). No significant associations were found between depression and DII, DI-GM, or CDAI (40), which may be due to sample characteristics, limitations of dietary assessment methods, or the complex etiology of depression. SHAP analysis highlighted key HEI-2015 components, underscoring the benefits of reducing added sugars and saturated fats while increasing whole fruit intake to lower depression risk (13, 41).

Although there is a theoretical basis supporting the association between DII and depression risk, our analysis did not find a significant relationship. Further examination of the distribution of DII components in our population revealed limited variability in the intake of certain key anti-inflammatory components (such as *ω*-3 fatty acids and flavonoids). Our subgroup analysis showed that while the total DII score was not significantly associated with depression, specific components such as refined grains and red meat intake were associated with increased depression risk (ORs = 1.24 and 1.31, respectively), while vegetable and fruit intake showed protective effects (ORs = 0.82 and 0.78, respectively). Regarding the null findings for DI-GM and CDAI, these may reflect limitations of these indices when applied without direct gut microbiome data and oxidative stress biomarkers, rather than indicating true biological null associations. Future studies should combine dietary assessment with microbiome sequencing and inflammatory/oxidative stress biomarkers to more comprehensively evaluate these mechanisms.

It is important to note that while our study employed multiple dietary indices to capture different aspects of dietary patterns (inflammatory potential, overall quality, gut microbiota influence, and antioxidant capacity), only HEI-2015 demonstrated significant association with depression risk after comprehensive adjustment. This finding suggests that overall diet quality, as captured by HEI-2015, may be more relevant to depression risk than these other specific dietary dimensions in our study population.

The superior performance of HEI-2015 in predicting depression risk likely stems from several factors. First, HEI-2015 provides the most comprehensive assessment of overall diet quality, incorporating both adequacy and moderation components. Second, HEI-2015 includes specific components such as added sugars and saturated fats (confirmed by our SHAP analysis as key factors), which were introduced in the 2015 version to replace the previous “empty calories” category, potentially capturing aspects of diet particularly relevant to mental health. Third, HEI-2015 evaluates consumption patterns of food groups rather than focusing on single nutrients or specific dietary mechanisms, providing a broader representation of dietary patterns that may affect mental health through multiple pathways simultaneously.

This study and the research by Wang et al. (8), both based on NHANES data, found a negative correlation between HEI-2015 scores and depression risk, though the effect sizes differed, primarily due to methodological variations. First, the two studies included different NHANES cycles, and population characteristics and dietary patterns may have changed over time. Second, this study employed more comprehensive adjustments for confounding factors, including metabolic parameters (blood lipids, glucose) and comorbidities (hypertension, diabetes, etc.), which may have partially attenuated the observed effect size. Third, this study utilized restricted cubic splines, mediation analysis, and machine learning methods to more deeply explore the nonlinear characteristics and mediating mechanisms of the relationship. Finally, differences in sample size may also have affected estimation precision. Despite variations in effect sizes, both studies consistently support a negative association between diet quality and depression risk, demonstrating the robustness of this relationship across different methodologies.

The bidirectional relationship between obesity (measured by BMI) and depression is well established. Obesity can elevate depression risk by promoting chronic low-grade inflammation, metabolic imbalance, and hormonal disruptions (42), leading to neuroimmune activation, neurotransmitter imbalances, and reduced cerebral nutrient supply (24). Conversely, depression can contribute to weight gain through lifestyle changes (such as overeating and inactivity) and neuroendocrine dysfunction, including heightened HPA axis activity (43). Our findings support BMI as a partial mediator in the diet–depression link, indicating that weight management is an important target for dietary strategies to prevent depression. Furthermore, reducing BMI may help relieve depressive symptoms by decreasing pro-inflammatory cytokines from adipose tissue, enhancing insulin sensitivity, and regulating bile acid metabolism, thereby conferring neuroprotective and metabolic benefits (44).

Dietary patterns are increasingly recognized as modifiable factors in depression prevention. In this study, four dietary indices HEI-2015, DII, DI-GM, and CDAI were assessed. Consistent with previous research (45, 46), higher HEI-2015 scores were linked to lower depression risk. Diets rich in vegetables, fruits, legumes, and whole grains may reduce depression risk by supporting endocrine function and neurotransmission through antioxidants (such as vitamin C and flavonoids) and anti-inflammatory fatty acids (e.g., *ω*-3 PUFAs). While the DII reflects the inflammatory potential of the diet and prior studies have related high-DII diets (high in saturated fats and sugars, low in fiber) to increased depression risk via neuroinflammation, no significant relationship between DII and depression was found here (47). Likewise, although DI-GM and CDAI are thought to influence depression through the gut-brain axis and by mitigating oxidative stress, no independent associations were observed, possibly due to the limitations of single dietary indices or confounding factors related to the multifaceted nature of depression (48).

The observed mediating effect of BMI on the relationship between HEI-2015 and depression suggests that weight management could be a valuable target for dietary interventions. Healthier diets may reduce depression risk both directly by improving metabolism and inflammation—and indirectly by lowering BMI and reducing obesity-related psychological and physiological impacts. Mediation analysis confirmed BMI’s partial mediating role, hinting that specific dietary elements (e.g., low-calorie, high-fiber foods) may impact mental health partly via weight and body fat reduction (49). Additionally, this study enhances mechanistic insights into how diet, BMI, and depression interact, and offers new perspectives for unraveling these complex relationships.

BMI may influence the relationship between HEI-2015 and depression through several pathways. First, healthier diets reduce body fat and chronic low-grade inflammation, a key contributor to depression especially in obese individuals (50). Lower systemic inflammation in turn lessens central nervous system inflammatory responses. Second, weight loss improves insulin sensitivity and increases brain-derived neurotrophic factor, supporting neurotransmitter function and reducing depression risk (51). Weight management can also enhance psychological well-being by boosting self-efficacy and social acceptance, further relieving depressive symptoms (52).

Our RCS analysis revealed a nonlinear relationship between HEI-2015 and depression, with the strongest protective effects observed at lower diet quality levels and a plateau effect at higher scores. This pattern suggests that initial improvements from poor to moderate diet quality may yield the greatest mental health benefits, while additional improvements from moderate to excellent diet quality provide more modest additional protection.

This nonlinearity has important implications for public health interventions. First, it suggests that resource-limited interventions might prioritize moving individuals from low to moderate diet quality, where the steepest reduction in depression risk occurs. Second, the plateau effect beyond approximately 65 points indicates that perfect adherence to dietary guidelines may not be necessary for substantial mental health benefits. Regarding analytical strategies, while the nonlinear relationship justifies consideration of alternative modeling approaches, we maintained linear models for several reasons: (1) the overall trend remained consistently protective across the HEI-2015 spectrum; (2) linear models provide more straightforward interpretation of effect estimates for clinical and public health applications; (3) quartile-based analyses confirmed the dose–response pattern; and (4) sensitivity analyses using fractional polynomial and spline-based models yielded qualitatively similar conclusions about the protective association. However, we acknowledge that future studies with larger sample sizes might benefit from more flexible modeling approaches to capture potential threshold effects more precisely. The observed nonlinearity may reflect biological mechanisms such as nutrient saturation effects or threshold phenomena in neurobiological pathways. Alternatively, it might indicate measurement limitations in capturing additional benefits of very high diet quality using current assessment methods.

SHAP analysis quantitatively identified the dietary components most relevant to depression. Reducing added sugar intake was crucial for lowering depression risk, consistent with prior studies connecting high-sugar diets to inflammation and mood disorders (53). Additionally, higher whole fruit consumption and lower saturated fat intake significantly strengthened the protective effect of HEI-2015. The antioxidants and fiber in fruits may help alleviate depressive symptoms by improving gut microbiota, reducing inflammation, and supporting neurotransmitter balance (54). These findings, quantified by SHAP, offer practical guidance for developing personalized dietary strategies for depression prevention (55). It is important to note that our SHAP analysis was deliberately limited to HEI-2015 components because this was the only dietary index showing significant association with depression. While we acknowledge that exploratory analysis of other indices’ components might have been theoretically possible, we elected to focus our machine learning approach on the significantly associated pattern to maintain methodological rigor and avoid potentially spurious findings from data mining non-significant associations.

This study has some limitations. Firstly, its cross-sectional design restricts the ability to infer causality between dietary patterns, BMI, and depression; longitudinal or interventional studies are needed for confirmation. Secondly, dietary assessment was based on two 24-h recalls, which may not accurately capture long-term habits and could compromise the reliability of DII, HEI-2015, DI-GM, and CDAI scores. Recall bias and subjective reporting may also affect data quality. Future research should use longer-term tools such as food frequency questionnaires or repeated recalls. Thirdly, although we adjusted for key confounders, some psychosocial and environmental factors were not fully considered. Fourth, depression was assessed via the PHQ-9, which measures symptoms rather than clinical diagnosis; future studies should include diagnostic criteria (e.g., DSM-5) or additional psychometric tools (e.g., HAM-D, BDI) for improved validity. Finally, the lack of biological data (e.g., metabolic markers, cytokines) limits insight into the mechanisms connecting BMI, diet, and depression risk.

## 5 Conclusion

Our mediation analysis indicates that BMI represents one potential pathway through which dietary patterns influence depression risk, highlighting the interconnected nature of nutritional epidemiology and weight management in mental health outcomes. Future interventions should consider evaluating both dietary modification and weight management components to better understand their distinct and shared mechanisms. SHAP analysis pinpointed important dietary components, providing actionable insights for depression prevention. Overall, these findings emphasize the value of combining dietary improvements with weight management to inform more effective and targeted nutritional psychiatry interventions.

## Data Availability

The original contributions presented in the study are included in the article/supplementary material, further inquiries can be directed to the corresponding author.

## Glossary

NHANES: National Health and Nutrition Examination Survey
PHQ-9: Patient Health Questionnaire-9
DII: Dietary Inflammatory Index
HEI-2015: Healthy Eating Index-2015
DIGM: Dietary Index for Gut Microbiota
CDAI: Composite Dietary Antioxidant Index
SHAP: SHapley Additive exPlanations
PIR: Poverty income ratio
BMI: Body mass index
HbA1c: Glycosylated hemoglobin
UA: Uric acid
BUN: Blood urea nitrogen
TG: Triglyceride
TC: Total cholesterol
HDL: High density lipoprotein
LDL: Low density lipoprotein
eGFR: Estimated glomerular filtration rate
ALT: Alanine aminotransferase
AST: Aspartate aminotransferase
CVD: Cardiovascular disorders
COPD: Chronic obstructive pulmonary disease
MLP: Multi-layer perceptron
DT: Decision tree
XgBoost: Extreme gradient boosting
LR: Logistic regression
RF: Random forest
